# Development and validation of a nomogram model to predict coronary heart disease in snoring patients with hypertension and non-glucose metabolism disorders

**DOI:** 10.5937/jomb0-57804

**Published:** 2025-11-05

**Authors:** Zhen Wei, Menghui Wang, Xiaoguang Yao, Liang Ming, Xintian Cai, Nanfang Li

**Affiliations:** 1 Hypertension Center of People's Hospital of Xinjiang Uygur Autonomous Region, Xinjiang Hypertension Institute; NHC Key Laboratory of Hypertension Clinical Research Key Laboratory of Xinjiang Uygur Autonomous Region "Hypertension Research Laboratory" Xinjiang Clinical Medical Research Center for Hypertension (Cardio-Cerebrovascular) Diseases, People's Hospital of Xinjiang Uygur Autonomous Region, Urumqi 830000 China; 2 Department of rehabilitation medicine of People's Hospital of Xinjiang Uygur Autonomous Region, Urumqi 830000 China

**Keywords:** snoring, hypertension, non-glucose metabolism, coronary heart disease, apnea index, nomogram, hrkanje, hipertenzija, metabolizam bez glukoze, koronarna bolest srca, indeks apneje, nomogram

## Abstract

**Background:**

Snoring, as a common disease, may lead to various cardiovascular diseases. scholars attach importance to the relationship between snoring or sleep breathing disorders and glucose metabolism disorders. Some predictive model for predicting sleep-disordered breathing in patients with diabetes has been developed. Studies have found that blood glucose is an inevitable predictor of the outcome of coronary heart disease. It may mask other predictive factors, leading to clinical neglect of the control of other risk factors. So we developed and validated a nomogram to predict the risk of coronary heart disease in hypertensive patients who snore, excluding those with glucose metabolism disorders.

**Methods:**

Records from 2105 snoring patients with hypertension and non-glucose metabolism disorders. A random grouping technique was utilized to split the data into validation and derivation datasets (split ratio = 0.7: 0.3). Least absolute shrinkage and selection operator regression was applied to select predictors and constructed a nomogram model based on multivariate Cox regression analysis. The discrimination and consistency of the nomogram were evaluated using the area under the receiver operating characteristic curve (AUC), calibration plots, and decision curve analysis (DCA) to assess its performance. We found age, male, waist-to-height ratio (WHtR), low and high-density lipoprotein cholesterol (LDL-C and HDL-C), and apnea index (AI) identified as predictors to generate this nomogram model.

**Results:**

The C-index at 84 months was 0.703 (95% confidence interval 0.653-0.754) in the derivation set and 0.645 (95% confidence interval 0.562-0.728) in the validation set. The nomogram demonstrated good performance in the calibration curve and DCA.

**Conclusions:**

So, our study proposed an effective nomogram model with potential application value for individualized prediction of coronary heart disease outcomes in snoring individuals with hypertension, excluding glucose metabolism disorders. And "AI" was proposed as a novel predictor.

## Abstract (sr)

**Uvod:**

Hrkanje, kao uobičajena bolest, može dovesti do različitih kardiovaskularnih bolesti. naučnici pridaju značaj odnosu između hrkanja ili poremecaja disanja u snu i poremećaja metabolizma glukoze. Razvijen je neki prediktivni model za predviđanje poremecaja disanja u snu kod pacijenata sa dijabetesom. Studije su otkrile da je glukoza u krvi neizbežan prediktor ishoda koronarne bolesti srca. Može da maskira druge prediktivne faktore, sto dovodi do kliničkog zanemarivanja kontrole drugih faktora rizika. Stoga smo razvili i potvrdili nomogram za predviđanje rizika od koronarne bolesti srca kod hipertenzivnih pacijenata koji hrču, isključujući one sa poremecajem metabolizma glukoze.

**Metode:**

Zapisi od 2105 hrkajucih pacijenata sa hipertenzijom i poremecajem metabolizma bez glukoze. Tehnika nasumičnog grupisanja je koriscena da se podaci podele u skupove podataka za validaciju i derivaciju (odnos podele = 0,7 :0 ,3 ). Najmanje apsolutno skupljanje i regresija operatora selekcije primenjena je za izbor prediktora i konstruisan je nomogramski model zasnovan na multivarijantnoj regresionoj analizi. Diskriminacija i konzistentnost nomograma su procenjeni korišćenjem površine ispod radne karakteristične krive prijemnika (AUC), kalibracionih dijagrama i analize krive odluke (DCA) da bi se procenio njegov učinak. Pronašli smo starost, muškarce, odnos struka i visine (VHtR), holesterol lipoproteina niske i visoke gustine (LDL-C i HDL-C) i indeks apneje (AI) identifikovane kao prediktori za generisanje ovog modela nomograma.

**Rezultati:**

C-indeks za 84 meseca bio je 0,703 (95% interval poverenja 0,653-0,754) u skupu derivacije i 0,645 (95% interval poverenja 0,562-0,728) u skupu za validaciju. Nomogram je pokazao dobre performanse u kalibracionoj krivoj i DCA.

**Zaključak:**

Dakle, naša studija je predložila efikasan model nomograma sa potencijalnom primenljivom vrednošću za individualizovano predviđanje ishoda koronarne bolesti srca kod osoba koje hrču sa hipertenzijom, isključujući poremećaje metabolizma glukoze. A "AI" je predložen kao novi prediktor.

## Introduction

Snoring is a prevalent condition, affecting approximately 20-40% of adults worldwide [1]. It is frequently associated with obstructive sleep apnea syndrome (OSAS), a disorder linked to various adverse health outcomes, including hypertension, stroke, coronary heart disease (CHD), and increased all-cause mortality [2] [3] [4] [5]. Meta-analyses suggest that habitual snoring alone is associated with a 28% increased risk of coronary artery disease (CAD), even in the absence of OSAS [6]. Some studies further indicate that snoring may independently contribute to cardiovascular risk, including the development of carotid atherosclerosis [7] [8].

Hypertension is a well-established risk factor for cardiovascular disease (CVD). A meta-analysis demonstrated that reducing systolic blood pressure (SBP) by 10 mmHg significantly lowers the risk of CVD events across all SBP levels [9]. When combined with snoring, the risk of CHD in hypertensive individuals is significantly amplified. Thus, early identification and intervention in snoring patients with hypertension may help mitigate the progression of CHD. Predictive models have been developed for such populations to estimate CHD risk [10] [11].

Additionally, increasing epidemiological evidence suggests a relationship between snoring and impaired glucose metabolism. Snoring has been associated with higher risks of impaired glucose tolerance and type 2 diabetes mellitus, particularly in the Chinese population [12] [13]. As a result, recent models have incorporated sleep-disordered breathing variables in diabetic cohorts to predict adverse cardiovascular outcomes [14] [15]. However, glucose metabolism is a dominant and unavoidable predictor of CHD outcomes. When diabetic and non-diabetic individuals are pooled together in prediction models, the predictive strength of blood glucose may overshadow other critical factors, potentially leading to clinical oversight in managing non-glucose-related risk indicators.

Given the limited evidence focusing exclusively on non-diabetic populations, we aimed to develop a CHD prediction model specifically for hypertensive snorers without glucose metabolism disorders. By excluding diabetic patients, we sought to identify alternative predictors and facilitate early screening and targeted intervention in this subgroup. This approach may aid in reducing the clinical burden of CHD and improving patient-specific outcomes.

To this end, we retrospectively analyzed a cohort of hypertensive snorers without glucose metabolism abnormalities and constructed a nomogram model. Nomograms provide a visually intuitive and clinically useful tool to quantify individualized CHD risk by incorporating multiple predictors into a single graphical interface.

## Materials and methods

### Study cohort

The information from our research, which includes historical data and follow-up details, was acquired from the catalog of a tertiary hospital. We examined the medical history of 3,065 hospitalized patients suffering from hypertension and snoring who were admitted to the Hypertension Center of the People's Hospital of Xinjiang Uygur Autonomous Region. All patients received hypertension-related assessments due to reported snoring from themselves or their family members at the Hypertension Center from January 1, 2010, to December 31, 2013. All patients had finished the polysomnography monitoring.

### Follow-up

Patients were followed for 1 to 10 years after discharge, with the endpoint of follow-up set as December 31, 2020. Follow-up data were collected through telephone interviews, outpatient visits, or hospital readmissions. Clinical outcomes were verified using medical records. Ethical approval for the study was granted by the Institutional Ethics Committee of the People's Hospital of Xinjiang Uygur Autonomous Region, and the requirement for informed consent was waived due to the retrospective design and anonymization of data. Exclusion criteria included: (1) presence of glucose metabolism disorders at baseline—defined as type 1 or type 2 diabetes mellitus, impaired glucose tolerance, fasting glucose >7 mmol/L, or use of hypoglycemic agents; (2) CHD events identified at baseline; (3) severe pulmonary disease; and (4) absence of physician-confirmed outcome data. After applying these criteria, a total of 2,105 patients were included. The dataset was randomly divided into derivation (70%) and validation (30%) cohorts.

### Data collection and definitions

Endpoint events: CHD was defined as coronary artery death, myocardial infarction and angina (including stable angina and unstable angina) [10]. CHD endpoint events were identified by two separate investigators who independently assessed outpatient visits, examined patients' medical records, conducted telephone interviews with patients, or reviewed death certificates. During the inclusion phase, we collected various demographic factors such as age and gender; anthropometric measurements that encompass body mass index (BMI), neck circumference (NC), waist circumference (WC), waist-to-height ratio (WHtR), and blood pressure levels, which consist of systolic blood pressure (SBP) and diastolic blood pressure (DBP); biochemical measurements that include high-density lipoprotein cholesterol (HDL), low-density lipoprotein cholesterol (LDL), triglycerides (TG), total cholesterol (TC), triglyceride-glucose index (TYG), fasting blood glucose (FBG), high-sensitivity C-reactive protein (hs-CRP), total cholesterol/high-density lipoprotein ratio (TC/HDL), triglyceride/high-density lipoprotein ratio (TG/HDL); and indicators obtained from polysomnography monitoring, which include the apnea-hypopnea index (AHI), apnea index (AI), hypoventilation index (HI), average blood oxygen saturation (Average SO2), lowest blood oxygen saturation (Lowest SO2), and smoking status categorized into (a) never smokers and (b) current smokers.

### LASSO regression

To prevent overfitting and select the most relevant predictors, the Least Absolute Shrinkage and Selection Operator (LASSO) regression was applied at the initial modeling stage. This method penalizes less informative variables by shrinking their coefficients toward zero, thereby simplifying the model while managing multicollinearity. LASSO regression was performed using the Lars algorithm in R (version 4.2.1). Variables identified by LASSO were then entered into a multivariate Cox regression model. Backward stepwise selection using Akaike Information Criterion (stepAIC) was applied to further refine the model. Comparative evaluation between the initial (Model 1) and optimized model (Model 2) was conducted using continuous net reclassification improvement (NRI) and integrated discrimination improvement (IDI) indices.

### Nomogram prediction model

A nomogram was constructed based on the final multivariate Cox regression model. Each predictor was assigned a point score according to its regression coefficient. The cumulative score corresponds to the estimated probability of CHD at the 84-month followup. To validate the model, bootstrap resampling was performed 1,000 times to assess internal stability. Discriminative ability was evaluated using time-dependent area under the curve (AUC) and receiver operating characteristic (ROC) analyses. Calibration was assessed by comparing predicted versus observed 7-year CHD risk. Decision curve analysis (DCA) was conducted to estimate clinical utility by calculating net benefit across different risk thresholds. All statistical procedures were performed using R (version 4.2.1) and STATA 15.0 (StataCorp, TX, USA).

### Statistical analyses

Missing values were addressed through multiple imputation using five datasets based on available covariates. The final dataset was derived by averaging imputed values, with any negative values replaced by the lowest plausible value. Continuous variables are reported as mean ± standard deviation or median (interquartile range), while categorical variables are expressed as frequencies and percentages. Patients (n=2105) were randomly allocated into a derivation cohort (70%) and a validation cohort (30%) using a random number generator. Univariate Cox proportional hazards regression was performed to assess the association between individual covariates and coronary heart disease (CHD) incidence. Variables analyzed included demographic, anthropometric, biochemical, and sleep-related indices. For categorical variables, reference categories were set as the first group. Hazard ratios (HRs) and 95% confidence intervais (CIs) were calculated, with statistical significance defined as p < 0.05. Variables with HR >1 were considered risk factors, while HR <1 indicated protective effects. All statistical analyses were performed using SPSS version 23.0 (IBM Corp., Armonk, NY).

## Results

### Baseline characteristics

**Figure 1:**
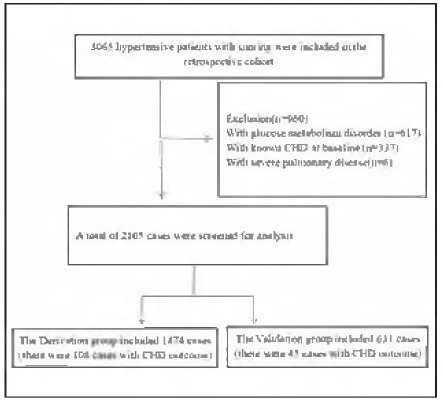
Flow Chart.

A total of 2,105 members were involved in this research, with 1,474 (70%) assigned to the derivation cohort and 631 (30%) to the validation cohort. Among these participants, 153 were diagnosed with CHD, representing 7.27% of the sample, and the average duration of follow-up period was 84 months. The median age at baseline for the 2,105 included patients was 46.63 ± 10.20 years, with an age range of 18 to 83 years. Figure 1 presents a flow diagram that outlines the design of the study. In our research cohort had missing information on NC (7.8%), WC (0.2%), TC (2.5%), TG (2.5%), HDL (2.6%), LDL (2.6%). Following the application of multiple imputations to fill in the missing data, there were no significant statistical differences observed in baseline demographics, clinical features, and indicators related to polysomnography monitoring between the validation and derivation cohorts. In the derivation cohort, there were statistically significant differences in several variables, including age, BMI, NC, WHtR, TC, LDL, HDL, TC/HDL, AHI, AI, Average SaO_2_, and Lowest SaO_2_, when comparing individuals with and without CHD. Similarly, in the validation cohort, significant differences were evident among the variables such as age, BMI, NC, WHtR, AHI, AI, HI, Average SaO_2_, and Lowest SaO_2_ between those with and without CHD. A summary of the baseline characteristics and differences in these variables across both the derivation and validation cohorts, based on the occurrence of CHD, is listed in Table 1.

**Table 1:** Clinical characteristics of the derivation and validation group; differences between the variables with and without cardiovascular heart events (CHD) of characteristics in the derivation and validation set. BMI: body mass index; NC: neck circumference; WHtR: waist-to-height ratio; HBP: high blood pressure; SBP: systolic pressure; DBP: diastolic pressure; TC: Total cholesterol; LDL: low density lipoprotein; HDL: high-density-lipoprotein; TG: Triglyceride; FBG: fasting-blood-glucose; hs-CRP: hyper-sensitive C-reative protein; TC/HDL: Total cholesterol/High density lipoprotein ratio; TG/HDL: Triglyceride/high density lipoprotein ratio; TYG Index: The triglyceride-glucose index; AHI: Apnea-hypopnea index; AI: Apnea index; HI: Hypoventilation inedxl; Averagr SO2 Average blood oxygen saturation; Lowest SO2 : Lowest blood oxygen saturation; CHD: Cardiovascular heart events.

	Derivation group		pa	Validation group		Pa	Pb
	With CHD<br>(108)	Without CHD<br>(1366)		With CHD<br>(45)	Without CHD<br>(586)		
Age (years)	50±11	46±10	0.001	49±11	47±10	0.115	0.270
Male (% (n))	81(75.0)	904(66.2)	0.061	33(73.3)	371(63.3)	0.177	0.214
BMI (kg/m[)	28.6±3.9	27.6±3.7	0.005	29.1±4.9	27.3±3.7	0.010	0.181
NC(cm)	40.8±3.4	39.6±3.8	0.001	40.6±3.4	39.4±3.8	0.031	0.285
WHtR	0.60±0.06	0.58±0.06	0.000	0.61±0.07	0.57±0.06	0.000	0.271
Smoker (% (n))	43(39.8)	431(31.6)	0.077	15(33.3)	170(29)	0.540	0.198
SBP (mmHg)	141±20	139±19	0.450	143±14	139±18	0.099	0.839
DBP (mmHg)	92±14	93±19	0.278	91±9	92±13	0.976	0.339
Laboratory examinations							
TC (mmol/L)	4.66±1.13	4.47±1.08	0.027	4.40±1.16	4.54±1.22	0.599	0.712
LDL (mmol/L)	2.84±0.84	2.61±0.74	0.004	2.67±0.90	2.63±0.79	0.702	0.854
HDL (mmol/L)	1.09±0.34	1.13±0.29	0.047	1.16±0.35	1.12±0.30	0.571	0.598
TG (mmol/L)	1.96(1.12,2.56)	1.96(1.15,2.23)	0.241	1.93(1.42,2.42)	2.01(1.19,2.35)	0.254	0.231
FBG (mmol/L)	4.35±0.62	4.30±0.67	0.502	4.40±0.65	4.35±0.70	0.362	0.078
hs-CRP (mg/L)	2.47(0.72,3.52)	2.52(0.80,3.40)	0.985	2.70(0.83,3.49)	2.52(0.82,3.46)	0.735	0.559
TC/HDL	4.57±1.45	4.18±1.77	0.001	3.97±1.00	4.25±1.33	0.331	0.305
TG/HDL	2.05(0.99,2.69)	1.96(0.97,2.25)	0.120	1.84(1.10,2.46)	2.03(1.02,2.41)	0.600	0.251
TYG INDEX	4.30(2.34,5.51)	4.24(2.41,4.85)	0.226	4.25(3.17,4.96)	4.45(2.52,5.04)	0.206	0.178
OSA measures							
AHI (events/hour)	22.78(5.90,30.88)	18.16(4.08,25.90)	0.022	20.64(8.20,33,20)	16.03(3.78,21.73)	0.006	0.061
AI (events/hour)	11.20(0.20,15.40)	16.66(0.00,7.00)	0.033	7.95(0.30,11.10)	5.75(0.0,4.93)	0.025	0.140
HI (events/hour)	12.14(4.18,18.08)	11.54(3.00,16.33)	0.210	12.74(5.85,17.75)	10.69(3.00,14.83)	0.038	0.349
Average SaO_2_ (%)	92±3	93±4	0.015	92±2	93±2	0.002	0.945
Lowest SaO_2_ (%)	79±10	81±9	0.013	79±9	81±8	0.013	0.901

### The Univariate Cox regression and Lasso regression

The association among each variable and CHD events in the derivation set was examined using Univariate Cox regression analysis. The univariable Cox regression analysis indicated that NC, Age, Male, Current smoking, LDL, HDL, TC/HDL, AI, and Lowest SaO_2_ were linked to the occurrence of CHD events. Lasso regression narrowed down the 22 variables to 9 potential predictors, which include Age, Male, NC, WHtR, Current smoking, LDL, HDL, TC/HDL, and AI. Furthermore, 22 variables were selected through the Lasso regression method, as detailed in Table 2.

**Table 2:** Univariable COX and LASSO regression analysis to extract the potential predictors respectively with CHD in the training set. BMI: body mass index; NC: neck circumference; WHtR: waist-to-height ratio; SBP: systolic pressure; DBP: diastolic pressure; TC: Total cholesterol; LDL,: low density lipoprotein; HDL: high density lipoprotein; TG: Triglyceride; FBG: fasting blood-glucose; hs-CRP: hypersensitive C-reactive protein; TC/HDL: Total cholesterol/high density lipoprotein ratio; TG/HDL: Triglyceride/high density lipoprotein ratio; TYG index: The triglyceride-glucose index; AHI: apnea-hy;popnea index; AI: Apnea index; HI: Hypoventilation index; Average SO_2_: Average blood oxygen saturation; lowest SO_2_: lowest oxygen saturation; CHD,: cardiovascular heart events.

Variables	Univariable Cox analysis		LASSO regression analysis
	HR (95% CI)	*P* value	Lambda =0.0096
Age	1.039(1.021,1.057)	0.000	0.026394123600852
Male	1.884(1.215,2.922)	0.005	3.13161658316703e-16
BMI	1.065(1.018,1.115)	0.007	0
NC	1.078(1.038,1.119)	0.000	0.0214663880855193
WHtR	39.866(2.523,630.023)	0.009	2.71689034659173
Current smoking	1.887(1.270,2.804)	0.002	-0.0260759495199308
SBP	1.000(0.990,1.099)	0.961	0
DBP	1.001(0.987,1.015)	0.894	0
TC	1.100(0.988,1.225)	0.081	0
LDL	1.499(1.188,1.890)	0.001	0.191403111281505
HDL	0.507(0.259,0.990)	0.047	-0.0200348350668604
TG	1.061(0.938,1.200)	0.349	0
FBG	1.077(0.814,1.426)	0.063	0
hs-CRP	0.991(0.917,1.071)	0.817	0
TC/HDL	1.056(1.010,1.103)	0.016	0.014909087038995
TG/HD	1.049(0.972,1.132)	0.223	0
TYG	1.026(0.974,1.081)	0.331	0
AHI	1.009(1.000,1.018)	0.052	0
AI	1.018(1.007,1.028)	0.001	0.00803162455337253
HI	0.997(0.981,1.014)	0.762	0
Mean SaO_2_	0.983(0.943,1.024)	0.408	0
Lowest SaO_2_	0.982(0.964,1.000)	0.047	0

### Multivariate Cox regression analysis in the derivation set

The dependent variable in this analysis was CHD events, while the independent variables identified through the LASSO regression method included nine potential risk factors: Age, Male, NC, WHtR, Current smoking, LDL, HDL, TC/HDL, and AI. After backward step by step selection in multivariate Cox regression analysis, Age (HR 1.04; 95% CI 1.02-1.06), Male(HR 1.67;95% CI 1.01-2.73), WHtR (HR 110.89; 95% CI 5.96-2026.11), LDL (HR 1.48; 95% CI 1.15-1.91), HDL (HR 0.51; 95% CI 0.24-1.11) and AI (HR 1.01; 95% CI 1.00-1.02), were bring into independent risk factors for CHD events. The constant IDI and NRI demonstrated a minor negative change from model 1 to model 2 (-0.000 and -0.070, p > 0.05; Table 3), but the change was not significant statistically. Therefore, Model 2 serves as a valuable predictive tool due to its simplicity and user-friendly design, requiring only six predictors. The findings of the multivariate Cox regression analysis are shown in Table 3.

**Table 3:** Step AIC Multivariate COX regression analysis of CHD selection to construct a nomogram model in the training set. NC: neck circumference; WHtR: waist-to-height ratio; LDL,: low density lipoprotein; HDL: high density lipoprotein; TC/HDL: Total cholesterol/high density lipoprotein ratio; AI: Apnea index.

Variables	Multivariate COX regression analysis					
	Model 1			Model 2		
	β	HR (95% CI)	P value	β	HR (95% CI)	P value
Age	0.04	1.04(1.02,1.06)	<0.0001	0.04	1.04(1.02,1.06)	<0.0001
Male	0.32	1.37(0.73,2.57)	0.3227	0.51	1.67(1.01,2.73)	0.0426
NC	0.02	1.02(0.95,1.10)	0.5286		-	
WHtR	4.13	62.35(1.98,1963.10)	0.0189	4.71	110.89(5.96,2026.11) 0.0016	
Current smoking	0.19	1.21(0.78,1.89)	0.1936		-	
LDL	0.38	1.46(1.12,1.89)	0.0047	0.39	1.48(1.15,1.91)	0.0022
HDL	-0.57	0.56(0.23,1.34)	0.1936	-0.07	0.51(0.24,1,11)	0.0983
TC/HDL	0.01	1.01(0.92,1.11)	0.8140		-	
AI	0.01	1.01(0.99,1.02)	0.0525	0.01	1.01(1.00,1.02) 0.0428	
*C-index <br>(95% CI) <br>^t^ IDI (95% CI) <br>^f^Continuous NRI <br>(95% CI)	0.706<br>(0.656, 0.757)				0.703 (0.653, 0.754) <br>-0.000(-0.012,0.001), p=0.259 <br>-0.070(-0.181,0.038), p=0.193	

### Establishment of a predicting nomogram

**Figure 2:**
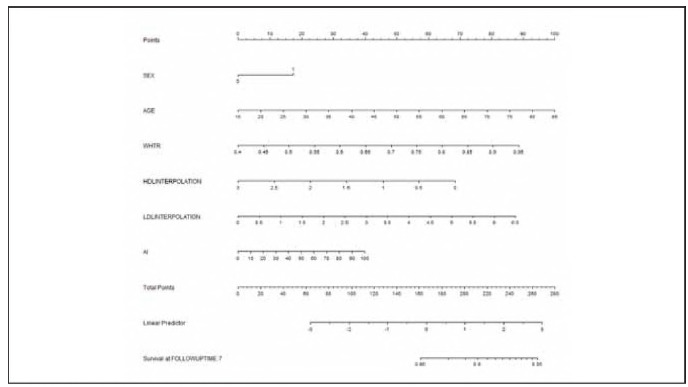
Nomogram model in the training set.

The nomogram was created to forecast the risk of a CHD event over 84 months in snoring patients who have hypertension and disturbances in non-glucose metabolism, relying on important predictors (Age, Male, WHtR, lDl, HDL and AI) in the derivation cohort (Figure 2). The value assigned to each individual was calculated based on the top Points scale, after which the points for each variable were summed. Ultimately, a customized 84-month risk of CHD events was determined based on the total points scale.

### Performance of the model for derivation and validation cohorts

**Figure 3:**
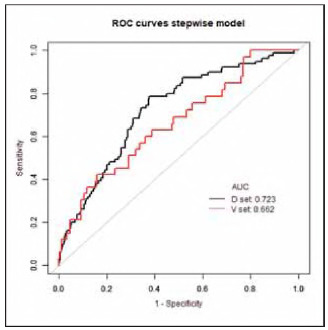
Comparison of ROC between derivation and validation set.

**Figure 4:**
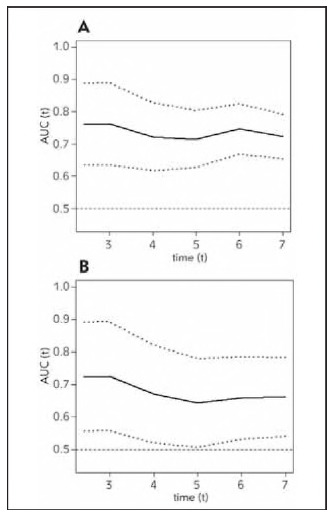
Time-dependent AUC in derivation (A) and validation (B) set.

The C-index for the nomogram predicting a personalized 84-month risk of CHD events was 0.703 (95% CI: 0.653-0.754) in the derivation cohort, whereas it was 0.645 (95% CI: 0.562-0.728) in the validation cohort. The area under the curve (AUC) for the derivation cohort was 0.723, while it was 0.662 for the validation cohort at the 7-year mark (Figure 3). Results from the time-dependent AUC analyses indicate that the time-dependent AUC consistently exceeded 0.6 in both the derivation and validation groups. These findings are illustrated in Figure 4A and Figure 4B, suggesting that the nomogram possesses strong discrimination and predictive capabilities.

**Figure 5:**
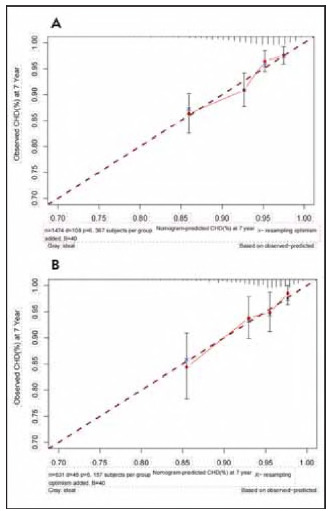
The calibration plots in the derivation set (5A) and validation set (5B) at 7 year.

**Figure 6:**
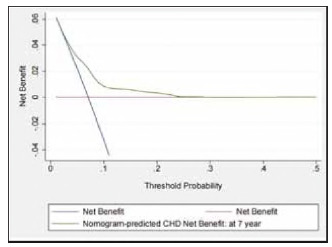
Decision curve analysis (DCA) for the Nomogram model. The green line represents the Nomogram model. The blue line represents the assumption that all patients occurred CHD, and the blue line represents the assumption that no patients had CHD. The Y-axis measures net benefifit. CHD, coronary heart disease.

The calibration effectiveness in both the derivation and validation groups was visually evaluated by charting the anticipated 84-month CHD event risk against the actual observed risk over the same period, as illustrated in Figure 5 and Figure 6.

## Discussion

In this study, we developed and validated a nomogram to predict coronary heart disease (CHD) in snoring patients with hypertension but without glucose metabolism disorders. Given that hyperglycemia is a well-established and dominant risk factor for CHD, its presence may overshadow other critical predictors, leading to insufficient risk management in non-diabetic populations. By excluding individuals with glucose metabolism disorders, we aimed to identify alternative risk markers that may otherwise remain undetected. The resulting nomogram demonstrated good calibration and discrimination, supporting its potential utility in clinical risk stratification and early intervention.

Our findings identified age, male sex, waist-to-height ratio (WHtR), low-density lipoprotein cholesterol (LDL-C), high-density lipoprotein cholesterol (HDL-C), and apnea index (AI) as independent predictors of CHD in this unique patient population. Among these, AI represents a novel predictor, reflecting the burden of obstructive sleep apnea (OSA)-related respiratory disturbances. Repeated apneas result in intermittent hypoxia, which promotes sympathetic activation, oxidative stress, endothelial dysfunction, and systemic inflammation [16]. These pathophysiological mechanisms contribute to cardiovascular injury. AI, as an indicator of apnea severity, reflects these risks and demonstrated predictive value comparable to or exceeding traditional lipid markers such as HDL-C.

The mechanical effects of obstructive events during sleep, including elevated transmural pressure across thoracic structures, may exacerbate myocardial workload, reduce stroke volume, and increase oxygen demand [17]. Although polysomnography (PSG) is the standard for diagnosing OSA, it is not widely implemented in routine practice due to resource limitations. However, AI could be estimated through clinical observation, providing a pragmatic tool for cardiovascular risk assessment in primary care settings. Given its weight in the nomogram, AI may serve as a surrogate for sleep-disordered breathing severity and should be considered in CHD risk screening among snoring individuals [18].

The decision to exclude diabetic patients stems from their high intrinsic CHD risk and the overwhelming influence of hyperglycemia-related mechanisms, including endothelial dysfunction, advanced glycation end products (AGEs), and chronic oxidative stress. In prior studies, diabetes has consistently emerged as a major determinant in CHD prediction models, including those applied to snoring and hypertensive populations [10] [11]. Its inclusion may obscure the contributions of other relevant factors. Our exclusion of diabetic individuals thus allowed us to uncover additional predictors specific to the non-diabetic subgroup.

Gender differences in CHD outcomes were evident in our cohort, consistent with previous epidemiological evidence. Men have a higher risk of CHD during their reproductive years, possibly due to hormonal differences, higher smoking prevalence, and poorer health behaviors [19]. In our analysis, male sex independently predicted CHD, reaffirming the importance of sex-specific considerations in cardiovascular risk prediction.

Anthropometric indicators such as WHtR have been shown to outperform body mass index (BMI) and waist circumference (WC) in predicting cardiometabolic risk. WHtR is simple, universally applicable, and less affected by ethnicity or body build. Our results further support WHtR as a robust, independent marker of CHD risk, consistent with the previous findings [20].

Dyslipidemia plays a central role in atherosclerotic disease. Elevated LDL-C facilitates arterial plaque formation through endothelial infiltration, oxidative modification, and subsequent inflammatory responses. Conversely, HDL-C exerts vasoprotective effects by promoting cholesterol efflux, suppressing inflammation, and enhancing endothelial function. Our nomogram incorporates both LDL-C and HDL-C, reinforcing their established relevance while demonstrating their continued predictive strength in a non-diabetic cohort.

### Limitations

This study has several limitations. First, its retrospective design and single-center data limit generalizability. Second, although AI is a meaningful variable, it may not be easily accessible in all clinical settings due to the reliance on PSG. Nonetheless, AI can be approximated through clinical observation, albeit with more effort. Third, the use of office blood pressure rather than ambulatory measurements might have underestimated the impact of nocturnal hypertension on CHD risk. Finally, while internal validation was performed, external validation in larger, multi-center cohorts is needed to confirm the model's applicability.

## Conclusion

We constructed and validated a nomogram to predict CHD in snoring, hypertensive patients without glucose metabolism disorders. This model incorporates both traditional and novel predictors, including AI, offering a practical and individualized tool for early CHD risk assessment in this specific population.

## Dodatak

### Declarations

### Approval of ethics and consent for participation

The study was approved by the Medical Ethics Committee of the People's Hospital in the Xinjiang Uygur Autonomous Region (Approval No. 2019030662) and conducted in accordance with the ethical principles set out in the Declaration of Helsinki and its subsequent revisions. Written informed consent was obtained from all participants or their legal representatives. The datasets generated and analyzed during this study are not publicly available due to ongoing research. However, requests for data can be made to the corresponding author with a valid inquiry.

### Consent for publication

By submitting my manuscript, I agree to pay the full Article Processing Charge if my work is accepted for publication (unless it is covered by an institutional agreement or journal partnership or a total waiver has been granted).

### Availability of data and materials

The data sets created or examined during this study are not available to the public; however, they can be acquired by reaching out to the corresponding author with a valid request.

### Conflicting interests

I confirm that the authors have no conflicts of interest, as specified by BMC. Additionally, they do not have any other relevant interests that could be perceived as affecting the integrity of the findings or the discussions presented in this paper. This statement underscores our commitment to transparency and objectivity in our research findings.

### Funding

This study was supported by Sub-project of Major Science and Technology Special Project of Xinjiang Uygur Autonomous Region (No.2022A03012-2).

### Authors' contributions

Zhen Wei wrote the main manuscript text, Nanfang Li, Menghui Wang design of the work; Xiaoguang Yao help interpreted the data; Ming liang and Xintian Cai responsible for data collection.

### Conflict of interest statement

All the authors declare that they have no conflict of interest in this work.
